# Molecular tools for bathing water assessment in Europe: Balancing social science research with a rapidly developing environmental science evidence-base

**DOI:** 10.1007/s13280-015-0698-9

**Published:** 2015-09-21

**Authors:** David M. Oliver, Nick D. Hanley, Melanie van Niekerk, David Kay, A. Louise Heathwaite, Sharyl J. M. Rabinovici, Julie L. Kinzelman, Lora E. Fleming, Jonathan Porter, Sabina Shaikh, Rob Fish, Sue Chilton, Julie Hewitt, Elaine Connolly, Andy Cummins, Klaus Glenk, Calum McPhail, Eric McRory, Alistair McVittie, Amanna Giles, Suzanne Roberts, Katherine Simpson, Dugald Tinch, Ted Thairs, Lisa M. Avery, Andy J. A. Vinten, Bill D. Watts, Richard S. Quilliam

**Affiliations:** Biological & Environmental Sciences, School of Natural Sciences, University of Stirling, Stirling, FK9 4LA UK; Department of Geography & Sustainable Development, University of St Andrews, St Andrews, KY16 9AL UK; Centre for Research into Environment & Health, Aberystwyth University, Wales, SA48 8HU UK; Lancaster Environment Centre, Lancaster University, Lancaster, LA1 4YQ UK; Policy Research Consultant, 1720 Le Roy Ave., Berkeley, CA 94709 USA; City of Racine Health Department Laboratory, 730 Washington Avenue, Racine, WI 53403 USA; European Centre for Environment & Human Health, University of Exeter Medical School, Truro Cornwall, TR1 3HD UK; National Laboratory Service, Environment Agency, Starcross, Devon EX6 8FD UK; University of Chicago, 5828 S University Avenue, Pick 121, Chicago, IL 60637 USA; School of Anthropology and Conservation, University of Kent, Canterbury, CT2 7NR UK; Newcastle University Business School, Newcastle upon Tyne, NE1 4SE UK; United States Environmental Protection Agency, Economic and Environmental Assessment Branch, Office of Science and Technology, Office of Water, Washington, DC USA; Department for Environment Food and Rural Affairs, Nobel House, 17 Smith Square, London, SW1P 3JR UK; Surfers Against Sewage, Unit 2, Wheal Kitty Workshops, St Agnes, Cornwall TR5 0RD UK; Land Economy, Environment & Society, Scotland’s Rural College (SRUC), Edinburgh, EH9 3JG UK; Scottish Environment Protection Agency, Eurocentral, North Lanarkshire ML1 4WQ UK; Scottish Environment Protection Agency, Stirling, FK9 4TZ UK; Environment Agency, Horizon House, Deanery Road, Bristol, BS1 5AH UK; Keep Scotland Beautiful, Glendevon House, Castle Business Park, Stirling, FK9 4TZ UK; Economics, Stirling Management School, University of Stirling, Stirling, FK9 4LA UK; School of Economics & Finance, University of Tasmania, Hobart, Australia; UK Water Industry Research Ltd, 8th Floor, 50 Broadway, London, SW1H 0RG UK; Environmental & Biochemical Sciences, James Hutton Institute, Craigiebuckler, Aberdeen, AB15 8QH Scotland, UK; Social, Economic & Geographical Sciences, James Hutton Institute, Craigiebuckler, Aberdeen, AB15 8QH Scotland, UK; Institute of Environment, Health & Societies, Brunel University, London, UK

**Keywords:** Bathing Water Directive, Fecal indicator organism, Microbial pollution, Public perception, Recreational water quality, Risk communication

## Abstract

The use of molecular tools, principally qPCR, versus traditional culture-based methods for quantifying microbial parameters (e.g., Fecal Indicator Organisms) in bathing waters generates considerable ongoing debate at the science–policy interface. Advances in science have allowed the development and application of molecular biological methods for rapid (~2 h) quantification of microbial pollution in bathing and recreational waters. In contrast, culture-based methods can take between 18 and 96 h for sample processing. Thus, molecular tools offer an opportunity to provide a more meaningful statement of microbial risk to water-users by providing near-real-time information enabling potentially more informed decision-making with regard to water-based activities. However, complementary studies concerning the potential costs and benefits of adopting rapid methods as a regulatory tool are in short supply. We report on findings from an international Working Group that examined the breadth of social impacts, challenges, and research opportunities associated with the application of molecular tools to bathing water regulations.

## Introduction

Regulation of bathing and recreational water quality is undertaken around the world to protect the environment, human health, and economic livelihoods (Nevers et al. 2014; Reder et al. 2015). Methods used to quantify microbial water quality vary but the most commonly employed approaches use nutrient rich media to culture fecal indicator organisms (FIOs, e.g., *Escherichia coli* and intestinal enterococci). The culture-based approach provides a widely used basis for informing on human health risks associated with sea-bathing via an established epidemiological evidence-base (Kay et al. 2004). The professional and regulatory norms that drive use of this technique are longstanding, and have developed in conjunction with investments in technological capacity and scientific infrastructures that make them cost effective and politically uncontroversial. However, culture-based methods are often criticized in terms of speed of analysis, which can take between 18 and 96 h for sample processing (Raith et al. 2014). With their potential to reduce sample processing time to between one and two hours, interest in molecular biological tools, such as quantitative polymerase chain reaction (qPCR), for beach management is growing. Briefly, qPCR is a nucleic acid-based approach that amplifies and simultaneously quantifies a DNA target. Thus, molecular biological tools offer an opportunity to provide a more meaningful statement of microbial risk to water-users by providing near-real-time information, enabling potentially more informed decision-making with regard to water-based activities (Mendes Silva and Domingues 2015).

The USA has begun to utilize qPCR methods at some recreational beaches on a voluntary basis. This was prompted by a lawsuit against the US Environmental Protection Agency (EPA) filed by the Natural Resources Defence Council (NRDC) and others which argued that USEPA had not delivered on its intention to explore new or revised water-quality criteria linked to rapid test methods (Gooch-Moore et al. 2011). In response, the USEPA published the 2012 revised Recreational Water Quality Criteria (RWQC) for FIOs in marine and fresh waters (U.S. EPA 2012); within these criteria there are regulatory action thresholds for enterococci as determined by qPCR. Individual states can choose to use these recommendations to make precautionary beach management decisions and/or to develop recreational water-quality standards in line with this approach (Haugland et al. 2014).

In general, the lawsuit is a good indication of institutionalized environmental agendas gradually fragmenting and changing in the light of new models of working although, in practical terms, the pattern of qPCR uptake and experimentation is uneven. While qPCR is now being used by some beach managers in the USA, the case for wide-scale adoption of this molecular approach is the focus of global debate, with regulators and researchers drawing attention to a number of technical and logistical issues associated with this emerging technology (Oliver et al. 2014). For example, the practical benefit of rapid analysis can be hampered, particularly in the EU, by centralized laboratory infrastructure (i.e., sample transit time to the laboratory exceeds the duration needed for qPCR analysis). There also remain uncertainties regarding the robustness of the qPCR epidemiological evidence-base (Oliver et al. 2014). In general, the prospect of international regulatory communities adopting qPCR as a “rapid” method, with defensible cost efficiencies, remains uncertain and long term.

The research community has invested significant effort in exploring the potential of molecular methods applied to beach management (Wade et al. 2006; Griffith et al. 2009; Whitman et al. 2010). While the evidence-base to underpin qPCR deployment for the regulation of microbial water pollution is expanding, it remains immature when compared with culture-based methods, and this limits the operational utility of qPCR within regulatory monitoring regimes outside the USA. Over time, these advances in research and development (R&D) will help to determine the viability of qPCR becoming a more commonly used tool for microbial water-quality management and regulation. There exist few complementary studies addressing the potential wider costs and benefits of adopting qPCR as a regulatory tool (Pratap et al. 2011). The likelihood of “win–win” scenarios versus “trade-off” scenarios concerning social-, economic-, environmental-, and health-related impacts resulting from rapid microbial water-quality reporting methods, relative to current approaches, remain largely anecdotal and need to be exposed to systematic and critical inquiry. Normative assumptions that flow from the case for rapid assessment need to be tested; such methods may also impact negatively on beach users as well as local communities and businesses reliant on tourism. With limited research documenting the importance of beach management decisions to a wider society (Rabinovici et al. 2004), the case for a coordinated research agenda, focusing on the wider social and economic impacts, and behavioral dimensions of “rapid methods” is arguably long overdue, and increasingly exposed as a policy research ‘gap’ given the pace at which environmental applications of qPCR have been developing. Others have also called for the emergence of research agendas that extend much further than ensuring water-quality standards alone are met (Bridge et al. 2010).

Moreover, if qPCR is adopted widely in the USA, as a preferred method for quantifying levels of fecal pollution, it is likely that exploring qPCR as a regulatory tool for enumerating microbial parameters under the EU Directives will emerge as procedural policy development need in the UK and the rest of Europe (Oliver et al. 2010). It was precisely in this context that an international Working Group (WG) was established in the UK under the auspices of the ‘Delivering Healthy Water (DHW)’ project. Within this a social and economic component of the WG was developed with the aim of interrogating and debating the existing evidence-base concerning wider social and economic impacts and complexities across local-to-regional-to-national scales, of a potential transition from culture- to molecular-based approaches for quantifying microbial compliance parameters in bathing waters. This commentary draws on the collective expertise of our international WG and spans policy, regulatory, non-governmental organization (NGO), and academic perspectives. It attempts to capture, for the first time, the breadth of opportunities and challenges that exist within this emerging social science research agenda concerning the scientific practices that inform and deliver on bathing water regulation.

## Emerging socioeconomic research themes

Our WG capitalized on extensive specialist and policy expertise associated with the participating members. The WG comprised 12 core members associated with the wider DHW project plus 16 experts spanning the disciplines of economics and social sciences. We augmented our WG further through an online-structured survey. This survey combined closed and open questioning and was embedded into the project website with a link which was distributed widely over a period of 2 weeks. This enabled us to capture global views on challenging, unresolved, or priority research questions from a cross section of academic, policy, regulatory, local authority, and NGO communities who accounted for 54, 6, 14, 3, and 23 % of responses, respectively. This process resulted in the collation of over 60 priority socioeconomic research questions and challenges associated with culture-to-molecular methodological transitions for evaluating bathing water quality. Using a workshop format, the WG distilled this information into six thematic groupings, removing duplication and ambiguity in the responses, and generating a record of priority research questions related to wider socioeconomic impacts of methodological transition (Table 1). Each thematic grouping of future research needs outlined in Table 1 is summarized below. Taken together, they describe a roadmap of social and economic research opportunities.

**Table 1:** Priority research questions

Direct cost implications	Types of information	How to measure & communicate risk	How to measure success of rapid methods	How to value a day at the beach & the cost of illness	Visitor behavior
What infrastructure & costs are needed to maximize the benefits of rapid methods?	What quantity & type of information would beach users prefer to receive? How quickly would they like to receive this information & how would they like to access it?	What information should be given to the public to allow more informed & better decisions about bathing water risk?	How would changes to the beach take shape (frequency/activities/indirect & direct economic impacts) should water-quality information be improved?	We need system-wide methods of assessment in order to understand the totality of benefits/trade-offs for valuations	What drives demand for beach use, how heterogeneous is it, & what role does water quality play in it?
Uncertainties in the scientific evidence-base hindering economic valuations need to be addressed	Does the preference for certain type of information or the way in which it is accessed vary between different user groups & if so how?	How can uncertainty regarding health risk be better incorporated into valuation scenarios of bathing water quality?	What are the additional (£/$) benefits in terms of enhanced ecosystem services from actions to reduce health risks in bathing waters?	Do we know enough about the vulnerability/WTP of different user groups with regard to health risks?	How do we distinguish the effects of changes in water quality compared to the effects of signs on beach-going habits?
How should investment be distributed between microbial risk management (beach monitoring) & prevention (catchment management)?	Would recreational water users react to information on water quality? What information would people respond to? What is the best way to present risk information, i.e., risk of GI infection	Is there a common set of demographic factors that explain variation in responses to risk information?	What are the measures by which we can (& want to) evaluate beach management success?	What are the economic impacts of illness as a result of exposure to polluted waters and how might rapid methods alter the cost of health-care?	Which groups of recreationists would be most affected by 1) advisory signs, 2) water-quality changes?
Would predictive modeling have more merit than using other methods requiring infrastructural reorganization?	Can we determine impacts on behavioral response of the same information being presented to recreational water users in different ways?	Does prediction of water quality have more value to beach users than “real” water-quality data?	How do we capture the benefits to new recreational water users who do not currently use a beach due to poor water quality?	Would the use of new methods lead to more beach failures? If so how would this change the value of a day at the beach? What would be the economic costs? What is the impact of posting warning signs at beaches to 1) users & 2) local economies?	What are the regional differences in attitudes & preferences regarding the impact of near-real-time water-quality information?

### Direct cost implications: investment, infrastructure, and logistics

From the perspective of economic cost, the potential to introduce and expand the use of molecular biological tools shares with any new technology and tool a burden of potentially high upfront investment costs. They require investment in training and associated infrastructures and require the expertise of more specialist staff. At the most basic economic level, moving from a cheaper (culture) method to a more expensive (qPCR) method will necessitate an initial phase of intensive training and infrastructure support followed by a period of concurrent monitoring and analysis via both culture and qPCR, i.e., to test equivalence, which would involve significant resource obligations at a time when finances available for environmental protection are limited (Griffith and Weisberg 2011). Automation and economies of scale could possibly reduce costs in the future. Additionally in the EU, modifying regional laboratory infrastructure would be a prerequisite for the deployment of qPCR as a method to quantify microbial compliance parameters in bathing waters. This is because the use of a national, centralized laboratory would only serve to undermine the benefits of rapid sample processing offered by qPCR, due to transport time.

The integration of geospatial and socioeconomic data via geographic information systems (GIS) could be utilized to determine the optimal spatial organization of regional laboratory infrastructure to enable a cost-effective and timely qPCR operation across the EU. However, the subsequent evaluation of potential infrastructure investments across EU member states would challenge individual governments. Infrastructure costs associated with the development of regional laboratories could certainly be estimated through tendering processes or market transactions, but the wider social and cultural benefits of such an investment are not normally directly valued in markets, thus creating a challenging (non-market monetary valuation) research opportunity for the determination of robust cost–benefit assessments (Hatton MacDonald et al. 2015). In addition, benefits arising from a shift to rapid assessment methods for bathing water regulation are likely to be long term and difficult to estimate, whereas the costs of bringing about this change of method deployment and any associated infrastructural reorganization would be much more immediate (Ostberg et al. 2012). Economic scenario modeling could provide a useful tool for exploring the potential value of benefits arising for future investment (see Table 1). For example, this might consider whether funds could be better spent on risk prevention (source water protection, i.e., catchment management) as opposed to risk management (i.e., beach monitoring and/or predictive modeling as recommended by WHO (2003)).

### Types of information: The what, when, and how

The use of scientific data to inform issues of water-quality and risk management varies considerably depending on the spatial and temporal scales at which problems and responses are being defined. Water assessment tools as instruments of policy delivery over the long term and at the macro-scale are defined by needs quite different to that of the micro-decisions of individual water user, situated and acting in a profoundly localized time and space. This creates a clear conflict in stakeholder preference for bathing water assessment tools. Regulatory compliance with the EU Bathing Water Directive (BWD) does not necessitate the use of rapid methods, and thus the speed of response is not a regulatory priority. In contrast, beach users are likely to welcome a more immediate “real-time” statement of the risk posed by bathing water quality in order to make better and more informed decisions concerning which beach to visit and what activities to undertake. The general question is whether our investments in science reflect these different purposes, and whether is it sufficient to align our preferences to one particular scale alone.

However, the underpinning debate is more complex for the alignment of science to micro-architectures of decision-making requires in and of itself a more challenging regime of scientific research. In the UK, for example, current monitoring regimes involve the collection of ~20 samples at each designated bathing water site during the bathing season. The adoption of a rapid method to inform on water quality would require an increased sampling frequency given that knowing quickly about microbial water quality is of limited value if sampling is only undertaken once a week. Indeed, evidence of significant within-day variability in bacterial compliance parameters at bathing beaches is growing suggesting that even daily sampling may not adequately characterize risk to bathers where the samples do not characterize the times of peak bather pressure (Mudd et al. 2012; Wyer et al. 2013). Before considering methodological transitions and the infrastructural reorganization necessary for regional laboratory analysis, and keeping in mind that regulatory compliance does not necessitate rapid sample processing, it is useful to situate innovation within a wider critical discussion of where priorities for knowledge generation and exchange reside. We might remark that the production of more information is not equivalent to the production of meaning from the perspective of beneficiaries and still less a guarantor of on-site behavioral changes and choices consistent with the minimization of risk. In the simplest terms, it is necessary to understand better what forms and types of information publics may need in order to act.

As highlighted in Table 1, little is known about how the public perceives the risk of illness associated with different microbial water-quality standards (e.g., risk of illness associated with ‘excellent’ versus ‘good’ versus ‘sufficient’ regulatory classifications of the BWD) or how this relates to a beach user’s acceptability threshold for FIO exposure during sea bathing (Pratap et al. 2013). Furthermore, this is likely to vary based on how the actual risk of sea-bathing is communicated to the public and whether bathers are visiting a beach environment singly or as part of a family group e.g., with children or immunosuppressed persons who may be more vulnerable to infection (Dufour et al. 2006; Wade et al. 2008; Pratap et al. 2013). And yet, in addressing these concerns, the issue is only partially a question of asking people what they want, which is generally an uncertain basis for understanding behavior, since the supposition is normative, and presumes, in any case, a priori requirements among “customers of information.” The issue is more about how the presentation and flow of information relates to conscious and unconscious patterns of in situ behavior; about understanding the ‘cues’ that automate, frame, and guide behaviors of different types of user. The normative basis of more rapid assessments directs science to questions of practical use and engagement with water bodies, rather than general progress against policy mandates operating over longer timescales, but we cannot presume that the provision of more rapid information would, in and of itself, be influential. This remains an open question (Hynes et al. 2009).

Questions over the form, timing, and method of providing rapid bathing water results would, therefore, represent clear priorities for future research. To avoid conflicting messages, on-beach presence would need consideration with respect to the EU wide bathing water classification symbols to be implemented at designated bathing waters at the end of the 2015 bathing season. The effectiveness of methods used to disseminate bathing water results are likely to vary across different bather communities reinforcing the need for near-real-time messages to be communicated in multiple formats rather than assume a “one-size fits all” approach to risk communication (Dearfield et al. 2014). In short, there is a need to instigate studies that understand better the way people act on relation to information, by testing alternatives in the context of a larger, and inevitably varied, user-behavioral narrative.

### How to measure and communicate risk

Public awareness of the potential environmental pollution and health impacts at beach environments is argued to be improving (Given et al. 2006). However, methodological transitions will bring a new set of public communication challenges requiring social science research that includes, but also is extended beyond, the matter of awareness raising, with its implicit assumption that influencing behavior rests largely, if not exclusively, on informing and changing people’s minds. The idea of awareness raising was certainly a common thread of debate among a group of international experts convened to debate the transitioning of new methods from R&D to an operational phase as part of the USA’s 2012 RWQC (Boehm et al. 2009). Carefully managed dissemination campaigns to inform beach users of how and why methods to evaluate microbial water quality have changed, and to highlight the strengths and limitations of such a shift in approach, would be necessary. New methods would also require the publication of health-based standards built on new epidemiological evidence and illness–response relationships, or as a minimum, proven equivalence at a range of sites between culture-based colony counts and qPCR gene copy numbers. The latter does not prove ‘viability’ of the indicator or related pathogens and this would need detailed attention particularly where effluents are disinfected with UV irradiance which can differentially attenuate culture-based and qPCR-based fecal indicators (Stapleton et al. 2009). Clarity and consistency in awareness-raising to inform people of the rationale behind such a methodological change to groups unfamiliar with the “culture versus qPCR” debate would be paramount.

In 2015, the EU BWD (2006) will have fully implemented a standardized classification system with associated pictorial symbols to reflect “excellent,” “good,” “sufficient,” and “poor” bathing water quality, as determined by culture-based methods. However, few beach users will be able to convert those classifications into a meaningful statement of risk with regards to the percentage chances of them becoming ill from exposure to bathing water. This is because little information is actually communicated to the public in terms of what a bathing water classification means with respect to health risk, and the inherent uncertainties associated with likelihood of illness (Pratap et al. 2013). Instead, the information tends to convey a “water quality communication” (Pratap et al. 2011). Furthermore, the demographic make-up of beach users receives little attention in terms of how information is communicated. For example, what is the best method to inform teenagers who might visit a beach environment unaccompanied by an adult and take little notice of traditional information displays of risks of sea-bathing or water-quality standards? Risk communication can be more effectively planned for and carried out if tailored to the particular audience and their current state of knowledge, behavior patterns, and attitudes about the risk issue (Weinstein et al. 1998). Translating such insights for bathing water management is an essential component of future research investment.

While the pursuit of speedier risk communication will continue to gather pace and necessitate a new epidemiological and social science evidence-base, it is time to pause and reflect on the quality and form of information currently provided to the public, and understand much more about how scientific data attains the status of information unlocking people’s capacities and inclination to act. Could improvements in risk communication offer a more effective approach to the management of microbial-related risks of sea-bathing relative to the deployment of qPCR? For example, it could be argued that publics might benefit more from being explicitly informed of the “percent chance” of illness from bathing at a particular beach rather than being told more quickly that bathing water quality is “sufficient” on any given day. However, there is ambiguity in how information would be interpreted—does a 5 % chance mean: (i) out of every 100 bathers 5 will become ill, or (ii) every 20 times a person bathes in water of this quality they are likely to be ill once?

A lack of attention has been given to how well the public recognizes the link between beach classification, measures of microbial pollution and recreational choices at bathing beaches. Little research has explored these questions, yet the answers would help underpin future efforts of effective beach management. Some may argue that communicating the percent likelihood of becoming ill is simply not necessary, shrouded in uncertainty given the differential susceptibility to illness across the population, and that ultimately any communication of measured water-quality data to the public will always be out of date to some extent given the potential for spatial and temporal variabilities in microbial compliance parameters (Mudd et al. 2012). Socioeconomic evidence also indicates that simple advisory messages about whether swimming is safe, or not, are given higher value than information on health risks (Eftec 2002).

This raises an interesting debate as to whether predictive modeling capability, as an alternative to laboratory-based “rapid methods” would offer the most “value” to beach users, irrespective of the form of information provision, because of its real-time nature. This is certainly an option and one that is being explored by the research and regulatory communities (Wyer et al. 2013). However, this too needs careful consideration of alternative communication challenges. Environmental modeling comes packaged with inherent uncertainties which may not necessarily be appreciated by members of the public. It is important to note, however, that bacterial enumeration by culture and molecular approaches is also subject to considerable enumeration imprecision (Environment Agency 2000).

### How to measure the success of rapid methods

Determining novel measures by which we can evaluate beach management success resulting from the use of more rapid methods offers prospects for exciting research. The most obvious measure in Europe is through the analysis of classification records under EU regulation; but how would the transition from culture to molecular-based approaches impact this measure of success? A sustained program of crossvalidation would be essential to prove statistical and public health equivalence with historical records (Oliver et al. 2010). However, a suite of social science methods could provide alternative interpretations and measures of the success of beach management against a backdrop of microbial water-quality results. For example, will there be changes in the frequency of visits and activities by the beach-user community if water-quality information is improved in terms of speed of provision? What would be the consequence of the use of rapid methods in one location for perceptions and uses of others? Given the competition for tourists across different coastal resorts, it is apparent that a rapid reporting of water quality (good or bad) could lead to a transfer of visitor spending into or out of local economies heavily reliant on holiday-maker visitation (Pendleton 2008) and in turn lead to water-quality measures becoming a “competitive weapon.” Tourism demand may then be further impacted by persistency and reputation effects of the positive or negative reporting of microbial water quality by rapid methods (Capacci et al. 2015). Little is known about the potential for additional economic benefits and wider social and cultural consequences arising from enhanced ecosystem services, ones that might be provided in response to the introduction of rapid measurement methods for more timely provision of bathing water-quality results and risk reduction. This seems to relate to a more general point about whether we are capable of collecting and combining evidence addressing the exceptionally wide variety of benefits and costs at stake for this issue. Intangible, long-term, and heterogeneous impacts are a particular challenge. Furthermore, success does not necessarily need to be measured against improvements in bathing water quality alone (Quilliam et al. 2015). Designated bathing waters and the surrounding beach environment provide cultural and ecosystem services linked to tourism and recreation that do not necessarily involve direct contact with the water environment, e.g., bird-watching and wildlife observation. Subsequently, numbers of beach users may locally increase in one location over another due to a perceived “better” environmental quality, irrespective of whether or not they are recreational water-users.

### How to value a day at the beach and the cost of illness

Many market and non-market monetary valuations of improvements in bathing water quality have been undertaken (e.g., Georgiou and Bateman 2005; Hynes et al. 2013). Also, being able to quantify costs and benefits of new sources of information allows decisions to be taken over how quickly and how widely to adapt this new technology, using cost–benefit criteria (Hanley and Barbier 2009). Such approaches provide a useful template for the exploration of the wider economic implications of a more rapid reporting of bathing water quality arising from methodological transitions. Other key questions relate to how qPCR-related classifications might affect tourism at coastal resorts, and the associated willingness of the public to pay for receiving rapid water-quality information. If rapid methods do inform the public better of an increased risk of illness on a given bathing day, beach users may not only be discouraged from going into the water, but also from visiting that particular beach altogether thereby reducing expenditure across that community. A relevant question is how this compares with the potential for healthcare savings and a reduction in the number of lost working days arising from more rapid water-quality reporting.

At the same time, and in pursuing these social and economic questions, we should not lose sight of gauging the significance and vagaries of these developments through qualitative study, rooted in the thoughts, and interpretations of those who ultimately bear these costs and benefits. There is a need to fully embed our science, and these questions of innovation, in their social and cultural worlds. Valuation analyses are just one part of the mix. Multiple studies exist on the wider use and non-use values from improved coastal water quality (e.g., Eggert and Olsson 2009; Ahtiainen et al. 2014). Similar studies designed to investigate the impact of “the need for speed” in bathing water-quality reporting are now warranted. Perhaps one of the most important social science research questions we need to ask is whether or not a more rapid reporting of bathing water quality, and the level of investment needed to deliver this effectively would actually deliver benefits over time that are large enough relative to the costs of changing to such a monitoring system?

### Visitor behavior

Understanding the spatial and temporal variabilities of beach-user habits across the UK (and indeed Europe and the rest of the world) will add additional layers of complexity for the academic community to consider in taking forward emerging research priorities in this field. Regional variations in local economic structures and beach-user attitudes and preferences are likely to impact on the perceived usefulness of rapid methods and the reporting of microbial water quality. Philosophically, it needs to be considered whether (and by how much) local preferences for different acceptable risk thresholds and cost–benefit trade-offs should play a role. Developing an understanding of how beach users perceive the provision of near-real-time water-quality information, and its implications for them personally and their overall community, is an important and novel area of research. However, changes to beach-going habits will be dependent on cognitive–affective processes (e.g., attitudes and motivation) inherently linked to past experiences (Brannstrom et al. 2015) as well as public perception of improved health outcomes associated with rapidly communicated bathing water classifications. Receptiveness of information will undoubtedly vary with audience (Pratap et al. 2013).

Some work has explored beachgoer’s views of other dimensions of beach management. For example, research exploring perceptions of the Blue Flag award scheme identified beach visitor age and type of stay as being influential in shaping public perception of this scheme, regardless of location (Lucrezi et al. 2015). This study also identified local residents as having greater awareness and knowledge of the Blue Flag scheme relative to visiting tourists; it would be interesting to investigate the parallels and contrasts in perceptions and understanding across different demographic groups with respect to any potential shift in water-quality assessment tools and reporting methods. Approaches to public participation, notably in the form of citizens’ juries, may offer opportunities for informing future decision-making in this area as well as the level of public understanding of the risks and uncertainties associated with the dissemination of water-quality information to beach users through the use of deliberative forms of environmental risk assessment (Fish et al. 2014).

## A ‘culture’ change in beach management?

Despite the scientific evidence-base, which is rapidly evolving in an effort to underpin the utility of qPCR for bathing water-quality assessment, there remain uncertainties over the desirability of wider deployment of this method for regulatory monitoring (Oliver et al. 2014). Efforts focus on the science and technology development with relatively little resources going into wider societal and cultural contexts and impacts (both positive and negative) concerning the use of “rapid methods” for informing the public about bathing and recreational water quality. It is understandable that significant effort has been invested in qPCR development with respect to bathing water science given the increased attention of method suitability in the US and a general interest in the value of rapid methods from the rest of the world. However, beach environments are also social environments, a source of well-being for beach users and others, and a key source of income revenue for some local economies (Quilliam et al. 2015). With that in mind, it is essential that the wider social ramifications of method transition are suitably explored to enable a coupling of science and technology developments with end-user needs and their assumptions and perceptions about risk and the cognitive and noncognitive behavioral contexts in which they are willing, able, or inclined to act. Currently, this coupling is weak because of a lack of socioeconomic and cultural investigation surrounding the potential impacts of qPCR deployment for quantifying microbial compliance parameters. Little appreciation of the breadth and extent to which methodological transitions might impact on wider society exists. Redressing this imbalance is now a priority, and one that offers considerable opportunity for interdisciplinary research.
